# Spontaneous Cure of Vaginal Hernia

**Published:** 1882-04

**Authors:** 


### SPONTANEOUS CURE OF VAGINAL HERNIA.

An extraordinary case of spontaneous cure of vaginal hernia, followed by perforation, has occurred in the service of M. Auger, of the St. Antoine Hospital. A woman, aged twenty-five years, mother of two children, became for the third time *enceinte*, and being displeased at this new pregnancy, consulted a midwife as to the best means of procuring abortion. Injection of tepid solution of coarse salt was advised and practised. The syringe employed had a long canula, and its introduction into the vagina caused considerable pain. Three injections were used, an interval of a week being allowed between each, and soon hemorrhage very abundant was excited. On the day of her entry into the hospital the patient, on sneezing, felt a tumor in the vagina, and soon it appeared at the orifice. Frightened, she came to the hospital, when, on examination, a tumor of a dark violet color, and resembling a loop of intestine, was observed between the labia. It was soft and seemed to contain gas. On exploring with the finger in the vagina the hernia was partially reduced, but it was impossible to find the point through which the intestine passed, neither could the os uteri be reached. However, no loss of substance could be detected in the recto-vaginal wall. There was no nausea or vomiting, the pulse was not accelerated, nor was the temperature lowered ; the abdomen was not tympanitic. Rest and constant poulticing were ordered. The following day the tumor was observed to be of a darker hue, which, toward evening, became still blacker and more salient. It also smelt gangrenous. The general health remained always undisturbed. On the afternoon of the third day the bowel burst, giving exit to hardened fecal matter, mixed with a blackish and fetid liquid, and a great quantity of gas. For two days fecal matter continued to pass through the artificial anus in abundance, but on the fourth day it had completely ceased, the bowels evacuating themselves through the rectum regularly. On passing the finger into the vagina no trace of the recent perforation could be found. Examination by the speculum gave no better results. On the twentieth day of her residence in the hospital the patient returned home in her usual health.—*Med. Press and Circular.*

